# A Case of Hæmorrhage from Inversion of the Uterus in Which the Operation of Transfusion Was Successfully Performed, with Remarks on the Employment of Transfusion Generally

**Published:** 1854-01

**Authors:** John Soden

**Affiliations:** Surgeon to the Bath General Hospital


### RECORD OF MEDICAL SCIENCE.

*A Case of Hæmorrhage from Inversion of the Uterus in which the operation of transfusion was successfully performed, with remarks on the employment of transfusion generally.* By John Soden, F.R.C.S., Surgeon to the Bath General Hospital.

I am impressed with a strong belief, that the extent of the evidence in favor of transfusion is very little known to the profession at large; that false notions prevail respecting its dangerous character, and the difficulties attending its execution; and that especially it does not receive justice at the hands of modern authorities in the practice of midwifery. It is not surprising that the extravagant results anticipated from its aid when first introduced by Lower and Denys,* should have disappointed the enthusiasm of its reception, and should have brought the operation into disuse here, and under the ban of an interdict in

* Soon after this operation was introduced at Paris, M. Denys performed it on five human subjects, two of whom recovered of disorders under which they labored; one, being in perfect health, suffered no inconvenience from it; and two persons who were ill, and submitted to the operation, died; in consequence of which the magistrates of Paris issued a sentence, prohibiting transfusion on human bodies under pain of imprisonment.

France ; but it is strange that this prejudice should still continue after the revival of transfusion by Dr. Blundell, upon a rational basis, founded upon extensive physiological experiment, and backed by the evidence so promptly and triumphantly testified to its value by Waller, Doubleday, and many others. My interest was first engaged in the subject of transfusion by an opportunity which occurred to me three years ago, of proving its power in the case which forms the subject of this paper. It appeared to me that the testimony of that case might be materially strengthened by combining with it the results of all the cases on record, and that possibly a sufficient number existed to afford such data as might lead to useful guides in practice, if not to establish positive laws for the employment of this operation. I found, however, that the instances of its general application were as yet too few, except in one class of cases, to admit of exact deductions ; I have therefore confined my Table to that exception, which relates to cases of hæmorrhage or exhaustion connected with the puerperal state,—and I think it will be heard with surprise, that the number of cases under this head that has come within my knowledge amounts to thirty-six. I was also influenced in not including in my list the whole field of cases to which this remedy is applicable, by finding in the course of my researches that my own idea had been already anticipated, and most ably executed, by Dr. Routh, in a Paper in the 'Medical Times' for August 11, 1849. Dr. Routh's investigation of the subject is so complete, and our conclusions so perfectly correspond, that I should have thought it unnecessary to have proceeded further, had I not been able to strengthen Dr. Routh's testimony by much valuable evidence that has accrued since the publication of his remarks, and also, I trust, to show the fallacy of the opinions generally entertained regarding transfusion.

### TABLE

OF

*Thirty-six Cases in which Transfusion was performed in consequence of Exhaustion or Hæmorrhage connected with the Puerperal State.*

| No. | Reference. | Particulars of Case. | Amount of Blood Transfused. | Result. |
|---|---|---|---|---|
| 1 | Blundel's Physiological Researches, p. 136. | Uterine hæmorrhage "during the birth of the placenta." Respiration had ceased for five or six minutes before transfusion was performed. Sixteen ounces procured from two men; readily injected. | 16 oz. | Unsuccessful. |
| 2 | —— | Uterine hæmorrhage "during the birth of the placenta." Respiration had not ceased, but the patient was in extremis. Three or four ounces only could be obtained from a lady. | 3 to 4 oz. | Unsuccessful. |
| 3 | Lancet, Oct. 8th, 1825; see also Waller's Book, 2d case. Mr. Doubleday. | Uterine hæmorrhage after delivery. Blood taken from the husband; when six ounces had been injected, the woman spoke. | About 14 oz. | Recovered. |
| 4 | Lancet, Nov. 19, 1825. Dr. Blundell and Dr. Uwins. | Violent flooding after the birth of the placenta. Transfusion performed four hours after hæmorrhage had ceased. Six ounces of blood were injected; this produced immediate improvement. Two hours after her powers again appeared to flag, and six ounces more were introduced. The blood was taken from two individuals. | 12 oz. | Recovered. |
| 5 | Lancet, March 4, 1826. Mr. Doubleday. | Uterine hæmorrhage. Woman cold and insensible. No particulars. | —— | Unsuccessful. |
| 6 | Lancet, April 29, 1826. Mr. Waller and Mr. Doubleday. | A female had been confined to bed for three weeks before labor, by severe vomiting, and was so reduced as to be unable to turn in bed without assistance. Shoulder presentation; delivered by turning. Hæmorrhage for five hours before delivery; collapse followed, and transfusion was had recourse to. | About 9 oz. | Successful. |
| 7 | Lancet, May 29, 1826. Mr. Ralph. | Abortion at the end of the third month. Copious hæmorrhage for ten hours, when Mr. Ralph was sent for. Transfusion performed eleven hours after the hæmorrhage had ceased. Four ounces only were injected; "in a few minutes animation was apparent." | 4 oz. | Recovered. |

| No. | Reference. | Particulars of Case. | Amount of Blood Transfused. | Result. |
|---|---|---|---|---|
| 8 | Lancet, Feb. 2, 1828. Mr. Clement. | Miscarriage, attended by violent hæmorrhage. Mr. Clement opened a vein in either arm, and injected about fifteen ounces from a stout, healthy man. "In a few hours she was perceptibly better," and gradually recovered. | About 15 oz. | Recovered. |
| 9 | Lancet, Feb. 9, 1828. Mr. Howell, Mr. Ravis, and Mr. Doubleday. | Transfusion before delivery. A woman had hæmorrhage at the commencement of labor; it ceased on the membranes being ruptured. Three hours afterwards labor-pains returned, and some degree of hæmorrhage; extreme prostration; pulse imperceptible. Blood obtained from the husband, a stout, healthy man. Fifteen ounces injected at intervals in fifty minutes. After five ounces had been introduced, the pulse became perceptible at the wrist; in another hour pains returned, and a dead child was speedily born. | 15 oz. | Recovered. |
| 10 | Lancet, Jan. 3, 1829. Dr. Blundell, Messrs. Davis, Pointer, and Lambert. | Flooding after the birth of the placenta. Eight ounces of blood transfused, at intervals, in the course of three hours. | 8 oz. | Successful. |
| 11 | London Medical and Physical Journal, February, 1827. Mr. Barton Brown. | In a tenth labor. Hæmorrhage came on before the birth of the placenta; the hand was introduced, and it, with the after-birth, was expelled by the uterine contractions. Flooding ceased, but the collapse continued; stimulants were freely administered, producing a partial rally. Three tolerably smart convulsive attacks at this time ensued, each followed by alarming collapse. Transfusion was had recourse to, two hours and a quarter after delivery. Thirteen drachms were first injected, without effect; after five minutes the same quantity was repeated, upon which the pulse began to return in the radial artery; in ten minutes a third quantity was injected, with increased signs of restoration; a fourth quantity was attempted, but abandoned from the restlessness of the patient. | About 5 oz. | Recovered. |
| 12 | London Medical and Physical Journal, July, 1827. Dr. Douglas Fox. | Uterine hæmorrhage followed the birth of a six months' fœtus; placenta retained by hour-glass contraction. Placenta removed, and the hæmorrhage ceased; but the patient remained in a state of extreme exhaustion. A teacupful of blood injected. | About 4 oz. | Recovered. |
| 13 | American Journal of Medical Sciences, 1830; from Journal Universel, March, 1820. | Uterine hæmorrhage at the third month of gestation. Transfusion successfully performed. | —— | Successful. |

| | | | | |
|---|---|---|---|---|
| 14 | Medico-Chirurgical Review, vol. viii, 1826. Mr. Waller's case. (See his pamphlet.) | Hæmorrhage after expulsion of placenta; perfect collapse; radial pulse just perceptible. Transfusion performed one hour after. Two ounces were injected, without effect; on repeating the amount, a tendency to syncope was observed, the pulse also filling a little, and some effort at vomiting was evinced. These symptoms ceased spontaneously, and the patient recovered. | 4 oz. | Recovered. |
| 15 | Medico-Chirurgical Review, vol. ix, 1826. Mr. Brigham. | Eighth pregnancy. Hæmorrhage three hours after the birth of the placenta. Five hours after commencement of the hæmorrhage evidently sinking. Two ounces of blood injected with a common syringe; after the third injection the pulse improved. Transfusion continued at intervals of ten or twenty minutes. | 10 to 12 oz. | Recovered. |
| 16 | Medico-Chirurgical Review. Mr. Jewell's case. | A delicate woman, exhausted by uterine hæmorrhage. No vein being discoverable in the arm, the external jugular vein was opened, and four ounces of blood were injected. No good effect followed, and the patient died. On a post-mortem examination, a small quantity of air was found in the right cardiac cavities; whether introduced by the injection, or the product of decomposition, doubtful. | 4 oz. | Unsuccessful. |
| 17 | Archives Générales de Médecine, vol. xxiv, 1830. M. Goudin, from Journal des Progres. | Hæmorrhage in the third month of pregnancy. Blood obtained from a healthy woman; four ounces injected, with a common eight-ounce syringe, two-thirds full. | 4 oz. | Successful. |
| 18 | Archives Générales, vol. iii, 1833. Dr. Banner's case. | Hæmorrhage from miscarriage, lasting twelve days. Transfusion was employed, with Blundell's apparatus. When five syringefuls had been injected, the breathing seemed oppressed; after a pause, two more syringefuls were thrown in, with increased oppression of the breathing. | Not named, but considerable. | Recovered. |
| 19 | Archives Générales, vol. vi, 1834. Dr. Klett. | Hæmorrhage in the second month of pregnancy. Blood taken from the husband; only two ounces injected. | 2 oz. | Successful. |
| 20 | Archives Générales, vol. vi. Dr. Klett. | Hæmorrhage for ten hours. Two ounces and a half injected. Taken from the husband. | 2½ oz. | Recovered. |
| 21 | Archives Générales, vol. xxv, 1851. M. Nitaton. | A woman, aged 20 had gone her full time; labor pains came on, with hæmorrhage, attributed to placenta prævia; delivered by turning. Ergot administered. Her condition rendered transfusion necessary. Three hundred grammes injected, by means of a hydrocele syringe. The cephalic vein was opened by a V shaped incision, and two ligatures placed round it, one to the distal side, to prevent regurgita- | 10 to 11 oz. | Successful. |

| No. | Reference. | Particulars of Case. | Amount of Blood Transfused. | Result. |
|---|---|---|---|---|
| | | tion of the blood injected, and the other to prevent the admission of air. The patient rallied immediately, and went on well to the seventh day, when abdominal pains appeared, and she died of puerperal peritonitis. | | |
| 22 | Gazette Médicale, 1838, p. 381. M. de Berg. | Ninth labour. Hæmorrhage after delivery, which continued at intervals for more than a fortnight, when transfusion became necessary. Two ounces and a half were injected by Dieffenbach's method. Resuscitation very gradual, but complete. | 2½ oz. | Successful. |
| 23 | Medical Gazette, vol. xiv. Mr. Bickersteth. | Delivery, at the eighth month, of the fourth child. Extensive interuterine hæmorrhage before delivery. In two hours afterwards transfusion performed, death appearing imminent. From ten to twelve ounces were injected; in less than two minutes the patient opened her eyes and showed signs of sensibility. | 10 to 12 oz. | Recovered rapidly. |
| 24 | Edinburgh Medical and Surgical Journal, No. 145, p. 40. Dr. Oliver. | A very important case. A woman, aged 42, attended by a midwife, with her seventh child. Violent hæmorrhage. Dr. Oliver performed transfusion about twelve hours after the delivery, the patient being in a state of perfect coma, and no pulse having been perceptible in the carotids for two hours and a half. The blood was supplied by three different individuals, and about twenty-two ounces injected gradually and at intervals. The first twelve ounces produced no effect, but recovery progressed as the injection was proceeded with. | 22 oz. | Successful. |
| 25 | Braithwaite's Retrospect, 1846; Northern Journal of Medicine, December, 1845. Mr. Brown. | A woman aged 37, liable to epilepsy. Suddenly attacked during labor with most alarming prostration. Craniotomy performed, and delivery effected. It does not appear that hæmorrhage took place, but no rally followed. Four ounces of blood were injected. | 4 oz. | Recovered immediately. |
| 26 | Guy's Hospital Reports, vol. ii, p. 256. | Placenta presentation; delivered by turning, by Mr. Lever. Great exhaustion from hæmorrhage; next morning, after exertion, the exhaustion returned, and threatened to be fatal at 3 P. M. Mr. Tweedie performed transfusion. Seven ounces were injected; the immediate effect was surprising, and animation was restored completely. In an hour afterwards relapse took place; Dr. Ashwell repeated the operation with the same effect, but not to the same degree, and more transient; she died in an hour afterwards. | 14 oz. | Unsuccessful. |

| | | | | |
|---|---|---|---|---|
| 27 | Dr. Collins's Treatise on Midwifery. | A patient in the Dublin Lying-in Hospital. In labor twenty hours, of her thirteenth child; pains severe during the greater part of the time. A quarter of an hour after the birth of the child, a sudden gush of blood took place, but not to a great extent. Dr. Collins introduced his hand, and found the uterus distended with blood; the placenta was readily detached, and the uterus expelled its contents, contracting well. No further hæmorrhage, but no rally took place. The same continued for ten hours; during that time two-thirds of a bottle of burned spirit and more than a pint of port wine were administered. Transfusion was then adopted; ten ounces were injected, which caused the woman to mutter indistinctly, but produced no other effect. The circulation was not improved, and she died in a few minutes. | 10 oz. | Unsuccessful. |
| 28 | Ingleby on Uterine Hæmorrhage. | Hæmorrhage after expulsion of placenta; prostration. Transfusion six hours after delivery! four ounces injected. | 4 oz. | Recovered rapidly. |
| 29 | Medical Times, Jan. 1848. Mr. Greaves; Dr. Waller. | Hæmorrhage in the eighth month of pregnancy. Flooding ceased; the vagina was filled with coagulated blood, which it was not thought prudent to disturb. No rally took place, and transfusion was had recourse to. Blood was drawn from a woman; when five ounces had been introduced, an amendment was evident; pulse more perceptible, and the countenance wore a better aspect. The blood now flowed sluggishly from the woman who supplied it. It was determined to wait; nourishment and stimuli were occasionally administered; after two hours and a half, the patient began again to sink, and jactitation supervened; four ounces more were again injected from the same individual, but this time without any effect. Blood was then drawn from the husband, a glassful of hot spirits and water having been first given him; the blood flowed from his arm "in an impetuous stream." The first injection of two ounces produced a marked alteration in the pulse; when nine ounces had been introduced the countenance was much improved, and after four ounces more all symptoms of danger had vanished. The case proved to be one of placenta presentation. After some hours' sleep labour came on, and the woman was safely delivered. | 22 oz. | Successful. |
| 30 | Lancet, March 28, 1835. Mr. Healey and Dr. Fraser. | A feeble strumous woman, aged 40. Uterine hæmorrhage, with retained placenta. Collapse had lasted six hours. Blood drawn from | 4 oz. | Recovered rapidly. |

| No. | Reference. | Particulars of Case. | Amount of Blood Transfused. | Result. |
|---|---|---|---|---|
| | | the husband, ("a strong healthy man;") four ounces transfused; rapid improvement; restoration complete in an hour. | | |
| 31 | Lancet, 1839–40. Mr. May. | Hæmorrhage from abortion, in the fifth month of pregnancy. The hæmorrhage had continued for some days, and an alarming condition for four hours. After eight ounces were injected, some rallying signs were observed, and improvement progressed till twenty-four and a half ounces had been transfused, when a slight turbulent action was observed in the carotids; it ceased immediately, and the patient rallied. The blood was supplied by four healthy males. In five days afterwards the bleeding returned, and she sank. | 24½ oz. | Successful. |
| 32 | Lancet, April 19, 1851. Mr. Masfen. | A delicate lady, aged 38, four months gone in pregnancy, fœtus having been dead two months; flooding; abortion. Transfusion performed two hours after insensibility became complete, blood supplied by a "stout buxom-looking servant-maid." Three ounces first injected; some degree of consciousness existed, but transitory; in half an hour a second three ounces injected, again followed by gradual relapse; after an hour a third injection was tried, with permanent restorative effect. Considerable inflammation followed in the vein below the elbow. Total quantity transfused, nine ounces, over a period of an hour and a half. | 9 oz. | Successful. |
| 33 | Lancet, December 13, 1851. M. Devay. | Transfusion performed at the Hôtel Dieu, Lyons, upon a young woman reduced to "mortal debility" by hæmorrhage, with successful result. Particulars not given. | —— | Successful. |
| 34 | Medical Times, Aug. 9, 1851, reported by Dr. Eve, from Gazette Médicale, July 5, 1851. Dr. Marmonier. | Case of flooding, after delivery by turning. Dr. Marmonier successfully performed transfusion, without any assistance, and with a child's toy syringe, holding about 70 grammes. The syringe was twice introduced, and about 90 grammes transfused. | About 3 oz. | Successful. |
| 35 | Dr. Crosse's Cases in Midwifery. | Patient aged 37. Placenta presentation. Great loss of blood had taken place for three weeks previously. When Dr. Crosse was called in, the patient was "pallid, exhausted, cold, pulse just perceptible." Delivery by turning, without additional loss of blood beyond what is usual. No rally took place. Transfusion performed to the extent of ten ounces, taken from the husband; death within an hour afterwards. | 10 oz. | Unsuccessful. |

Case 36.—On January 7th, 1849, a lady was attended in Bath by Mr. Ormond, a gentleman of great experience and intelligence, at the birth of her second child. The labor was rapid, and the latter pains so severe that the uterus was violently emptied of its contents and became inverted; a gush of blood ensued, and the patient fainted—the placenta was detached and the uterus returned to its natural situation. No further hæmorrhage took place—I joined Mr. Ormond in about half an hour—the patient had not rallied—she was lying on her back, insensible, perfectly cold, pulseless, and exsanguine in appearance—the only signs of life were short, jerking respirations at long intervals, and of a stertorous character. Nothing could look more unpromising than her condition at that time. Mr. Ormond was using means to restore warmth by friction, mustard-poultices, &c., and was administering brandy by teaspoonfuls, which the patient was just able to swallow. These means had now been employed for more than half-an-hour without any effect, and there scarcely seemed any hope that the patient would recover from the extreme exhaustion in which she then lay. We agreed that transfusion was the only resource, and I left Mr. Ormond to procure the instrument that occurred to me at the moment as best adapted for the purpose. I returned in half an hour, and during the interval Mr. Norman had arrived. No change had taken place in the patient's condition; and as we were now prepared to act, we determined to wait a little longer to watch for any indication of the course the case would take. This was presently afforded in an unmistakeable manner. The patient was no longer able to swallow, the respirations became more rare and stertorous, and were evidently on the point of ceasing altogether. Transfusion was now had recourse to, and the following plan was adopted for its execution. My instrument was a well-made syringe of German silver with a detached stop cock, it was larger than was desirable, being capable of holding seven ounces. Mr. Norman exposed the external cephalic vein, by an incision two inches in length, (the arm being fat.) Mr. Ormond bled the husband, the blood being received into a small deep basin, standing in another containing hot water. As soon as sufficient blood had been drawn, I filled the syringe previously well warmed and invested with a hot cloth, and at once proceeded to inject the blood into the vein. At first it would not pass up, but returned by the side of the pipe; presently the opposition from the close contact of the coats of the vein seemed to give way, and the blood, though impelled by a steady and moderate pressure, rushed up the vein with a rapidity that the eye could scarcely trace. The effect was electrical; instantaneously a convulsion seized the whole frame, and the muscles of the face were frightfully distorted. I paused in the injection, and I do not think more than an ounce could have found its way into the circulation; happily it was sufficient: the convulsion was but momentary, and signs of returning animation immediately succeeded. A restless movement pervaded the whole body—the arms were tossed over the head, and though consciousness did not return, the patient faintly but audibly spoke, muttering two or three times the expression "so tired," "so tired," she seemed to pass from a state of coma into one of syncope. The heart's action was now distinctly perceptible, and the vital energy gradually but very slowly returned : it was full an hour before any pulse could be felt at the wrists, and though the recovery steadily progressed without relapse, the patient did not recover consciousness until the following morning. During the whole of this time, every means was used to promote warmth, and no difficulty was experienced in getting the stimulants swallowed, that were from time to time administered. Some inflammation was set up in the fore arm below the point of the incision, but it was not of any moment, and subsided in two or three days, with the application of fomentations only. I hear from Mr. Ormond, that the patient remained for a long time in an exsanguine state, and complained of weakness and pain in the back. She did not nurse her child, and the catamenia returned in three months. After this period she left for change of air, and Mr. Ormond lost sight of her, but he subsequently heard, that, suffering from leucorrhœa, she went to London, and was treated for ulceration of the womb. This lady is now in India ; recent accounts have been received of the birth of another child and the well-doing of the mother.

The details of this case exemplify most of the important points connected with the performance of the operation. The rapid and decisive results obtained are by no means singular ; on the contrary, the narrators of many of the cases in the Table show parallel success, and describe in forcible language the wonder and surprise excited in their minds by the almost miraculous resuscitations they had witnessed. An analysis of the above Table shows that out of thirty-six cases, twenty-nine were recovered from imminent death by the operation of transfusion, and in seven only its performance was unsuccessful in restoring animation. It does not appear that the fatal termination in any case was due to or hastened by the operation, though in two instances, the latter effect was, on no warrantable grounds, attributed to its influence.

Of the seven fatal cases—in No. 1, respiration had ceased before the operation was performed, and the injection of twelve ounces produced no effect ; in No. 2, the failure was owing to the insufficient supply of blood ; in No. 5, it is probable that the same cause operated as in No. 1 ; in No. 16, no effect was produced. No. 26, occurred at Guy's Hospital : when seven ounces had been injected, the immediate effect was surprising, and animation was completely restored ; after an hour, relapse took place, and a second injection, by Dr. Ashwell, was attended by a similar reanimating effect, but in a less degree, and more transient ; the patient died in an hour afterwards ; the case had been one of placenta presentation, and the exhaustion was very great from long-continued hæmorrhage. No. 27 is an important case. It is narrated by Dr. Collins, in his treatise on Midwifery, and occurred during his Mastership at the Dublin Hospital. The circumstances of the case suggested transfusion as the only resource. The remedy, I conclude, did not stand in higher favor with Dr. Collins, than it experienced from most of the authorities in his branch of the profession ; and the unfavorable result seems, without sufficient grounds, to have strengthened his prejudice against it. A woman, in a very debilitated condition, and reduced by continued exposure to hardship, was admitted into the hospital, in labor of her thirteenth child. "She was not delivered for twenty hours, during the greater part of which time her pains were very severe: her child was born alive. Fifteen or twenty minutes after, a dash of blood suddenly took place from the uterus, not, however, to any unusual extent; the after-birth had not yet been thrown off." This was removed, and the uterus contracted well: no more blood was lost. The syncope, however, continued, in spite of an enormous quantity of stimulants, and was allowed to go on for ten hours; then, at the last gasp, transfusion was had recourse to, and "it did not seem to have any more marked effect than that of causing the woman to mutter indistinctly; the circulation was not improved, though we injected about ten ounces of blood. She expired within a few minutes after the operation." Dr. Collins thinks, though he anticipated her death, that it was hastened by the transfusion. No. 35: this case is equally important. Dr. Crosse, under whose hands it occurred, like Dr. Collins, attributes an injurious influence to the transfusion. The operation was certainly unavailing; but, considering the circumstances of the case, the general evidence on the subject, and the fact, that no especial symptom was developed, marking its connection with the transfusion, his conclusion seems hardly warranted. A woman, aged thirty-seven, had a placenta presentation; a great loss of blood had taken place at intervals for three weeks previously. When Dr. Crosse was called in, the patient was "pallid, exhausted, cold, with a pulse just perceptible;" delivery was speedily accomplished by turning, and without additional loss of blood beyond what is usual; no rally took place; transfusion was performed to the extent of ten ounces, taken from her husband's arm. Whilst this was doing, the patient became more distressed, "the pupils dilated, purplish pallor of the face, pulse no longer perceptible, death within an hour after." Thus of the seven fatal cases, in two it may be presumed that the women were dead before the operation was performed; in a third, only a small quantity of blood could be procured, and that from a weakly woman; in the fourth, no effect; in the fifth, the first effect of the transfusion was surprisingly restorative, but it was not permanent; in the sixth and seventh cases, of which I have fully given the particulars, the women were too much reduced to be restored,—the one, by too protracted a labour and subsequent collapse; the other, by repeated draining. I think it may be fairly urged that death was inevitable in each of these cases, and that in no instance was it attributable to, nor were any ill consequences produced by the transfusion. Transfusion is not to be regarded simply as the restoration of a certain deficiency of blood; for the benefit it affords bears but a trifling relation to any rule of loss and supply; nor is its agency to be attributed solely to its mechanical influence, as a warm fluid upon the heart. The transfusion of human blood may claim to be a direct, powerful,—I might almost add, when applied early,—unfailing stimulus to exhausted energy, even at the lowest point of existence, and when past the restorative aid of any other known means, either extraneous or inherent. The rapidity of the revival, however, varies; it depends not only on the circumstances of the case, as regards the previous duration and cause of the exhaustion, which, as is shown by the Table, exerts a material influence upon the power of the remedy, but also upon the character of the means used. Under this head, I include the quantity of the fluid to be transfused, and the mode of performing the operation. Inattention to these three points, was, perhaps, the cause of failure in five of the seven unsuccessful cases.

From the Table it appears that the total *quantity* transfused varied exceedingly; the lowest amount named is from one to two ounces; the highest, twenty-four ounces and a-half. The intermediate quantities varying a good deal, but with a preponderance to the low amount.

| | | | | | |
|---|---|---|---|---|---|
| 1 oz. | was injected in | 1 case, | 10 oz. | were injected in | 4 cases, |
| 2 oz. | " | 2 cases, | 12 oz. | " | 1 case, |
| 2½ oz. | " | 3 cases, | 14 oz. | " | 2 cases, |
| 4 oz. | " | 7 cases, | 15 oz. | " | 2 cases, |
| 5 oz. | " | 1 case, | 16 oz. | " | 1 case, |
| 6 oz. | " | 2 cases, | 22 oz. | " | 2 cases, |
| 8 oz. | " | 1 case, | 24½ oz. | " | 1 case, |
| 9 oz. | " | 2 cases, | Quantity not named in | | 4 cases.* |

No mean can be taken from these data to afford a practical guide—the quantity required was determined by the peculiar circumstances of each case; but generally, it may be said, that the lesser amount was needed in proportion as the exhaustion arose from the suddenness of the shock rather than from the extent of the hæmorrhage, and the cases most difficult of revival were those of placenta plævia, where the system had been reduced by repeated returns of hæmorrhage before labor. In Case 36, the heart responded immediately to the application of the stimulus. In Case 24, where the hæmorrhage had been violent, and the coma complete, no pulse having been perceptible in the carotids for two hours and a half, as much as twelve ounces was injected without any effect being produced, but recovery gradually progressed as the injection was proceeded with. It does not appear that too much blood was ever injected, or that any signs presented themselves indicating repletion. A large quantity of fluid may be introduced into the system without ill effect. In cholera, more than one hundred ounces of saline injection were sometimes used. Among the prejudices against transfusion, the fear of serious consequences from engorgement of the brain or heart, by the injection of too large a quantity, has been injuriously influential,—on some occasions the injection was not pushed to the extent of producing any effect; in such cases its power was not tested at all, and life was probably sacrificed by the over-caution of the operator. The evidence regarding the *quality* of the fluid transfused is very conclusive as to its importance. The propriety of selecting a good subject to supply the blood has been anticipated, and strongly inculcated by most of the advocates of transfusion; but experience has shown, that this is of more consequence than could have been expected. In the early days of trans-

* The quantity employed in each of the seven unsuccessful cases in this Table was as follows: in two, 4 oz.; in two, 10 oz.; in one, 14 oz.; in one, 16 oz.; and in one, unknown.

fusion the blood of animals was frequently employed;* and even recently the blood of a goat was successfully transfused in a case of hæmorrhage from phthisis. With the more accurate knowledge which we now possess of the physical distinctions between human blood and that of animals, such an example, though it was successful, is not likely to be followed, and the objections to it are too obvious to need recital. When transfusion was expected to work the miracle of perpetuating youth and curing all diseases, a variety of substances, such as gum, camphor, &c., was introduced into the veins; the failure of these experiments soon brought the remedy into disfavor. The idea, however, of the transfusion of artificial substances is not altogether to be condemned. It appears that the blood transfused may be drawn from different individuals with equally good effect, as from one alone, and that no evil results from the combination. In Case 31, where 24½ ounces were injected, the blood was drawn from "four healthy males;" in No. 4, from two individuals; in No. 24, from three; in No. 29, from two,—and this case is important, as showing the value to be attached to the character of the blood. The blood transfused was first taken from a weakly woman: when five ounces had been injected an amendment was evident, and the pulse became perceptible; as the blood now flowed sluggishly from the woman who supplied it, it was determined to wait; after two hours and a half the patient again began to sink and jactitation supervened—four more ounces were again injected from the same individual, but this time without any effect. Blood was then drawn from the husband, a glassful of hot spirits and water having been first given him; the blood flowed from his arm "in an impetuous stream;" the first injection of two ounces produced a marked improvement.

Regarding the *mode of performing* transfusion, I have come to the usual conclusion, that the most simple means are the best. No instrument is so convenient or safe for the purpose, as a common syringe. It should be accurately made, fitted with a detached stop-cock, plated or tinned on the inside, and capable of holding about three ounces. An infinite variety of apparatus has been invented for transfusing, not so much with the object of facilitating its performance, as of guarding against the danger of admitting air with the fluid injected; a danger that is nearly imaginary, and not founded on any ground of probability. An impression of this danger so generally exists in the profession, that it has been the greatest obstacle to the more frequent employment of transfusion. In many of the cases cited, the instrument made use of is not mentioned; sometimes a special apparatus was employed, and often a common syringe. No difficulty appears to have been experienced from the nature of the instrument used, except in one instance, and in that case the special apparatus was changed for a simple syringe. Dr. Marmonier, Case 34, is said to have used a common child's toy syringe; and great credit is due to him, that with such simple means, and without assistance, he undertook and successfully performed the operation. In the account of Case No. 36, I have stated the impediment the empty state of the vein seemed

* Journal de Pharmacie, May 1842. Dr. Blieding.

to offer to the entrance of the blood, and that on the opposition being overcome the wave of blood was traced with great rapidity towards the heart. It is well known that the danger of air finding admission into the veins is greater in operations about the neck than in those upon the extremities. I do not imagine that this circumstance depends upon the greater size of the veins, or their contiguity to the heart, as in the former case, but on the mechanical cause of the different incidence of the atmospheric pressure. A vein when divided remains empty for a considerable distance above the point of division, and the air is prevented from entering by its own external pressure along the trunk; by this means, the coats of the vein are maintained in contact. In the neck this does not so well apply; the external pressure can there only fall upon the orifice, when the division is low down, and not upon the trunk beyond; consequently, there is less opposition to the suction of the air into the vacuum of the vein: on this account a vein in the neck ought never to be chosen for the operation. In all the cases in the list, the usual situation of venesection was adopted, except in one instance, No. 16, where the external jugular was selected, from there being no apparent vein in the arm; the patient died in an hour after the transfusion. As the presence of air was on a post-mortem examination, demonstrated in the heart, a doubt existed whether it was attributable to the injection or to decomposition. No circumstances occurred at the moment of transfusion to indicate that the accident had happened; and where it has taken place in the division of veins during an operation, it has always declared itself,—sometimes by an audible sound with which the air rushes into the vacuum. In performing transfusion there is but one source of danger from this circumstance, and strangely enough, though it is the most obvious, it has been entirely overlooked by the different inventors of special apparatus. Many of their instruments are ingeniously calculated to promote the very object they are designed to prevent, namely, the passage of air with the blood through the instrument, and none of them offer an equal security, on this point, to a common syringe when used with proper and obvious precautions. The syringe has also the same advantage against the only chance through which the air might gain admission into the vein, in a dangerous form, for a few bubbles injected with the blood are unimportant, and that is, its passage, by the side of the instrument, into the empty vein, when the coats of the latter are separated by the introduction of the pipe; pressure beyond the opening till the blood is emerging from the syringe, would effectually guard against this possibility, or by the injection being performed under water.

In conclusion I will briefly remark on the general applicability of this remedy. An equal success has attended its adoption in the exhaustion caused simply by loss of blood under any circumstances. It has been chiefly tried after wounds and operations, of which Dr. Routh gives many examples. Its power is equally great where the diminished blood in the system depends upon inanition, as upon direct loss. A very valuable case illustrating this point has been published by the late Dr.

Pritchard and Mr. Clarke.* The case was one of atrophy from dyspepsia and constant vomiting. Sixteen oz. were injected, and the patient instantly revived, and completely recovered in three months. Transfusion has been of temporary service even where organic disease has been present, as phthisis and cancer of the stomach. Where an animal poison exists in the system in an active form, it has not been successful, as in hydrophobia, where it failed in the hands of Dieffenbach and Magendie; but this is almost an untried field. Dr. Routh especially alludes to the benefit that might be expected from its employment in the collapse of typhus fever. I witnessed its trial in a case of this description, several years ago, by Dr. Stokes, at the Meath Hospital. The patient was a middle-aged woman. Eight or ten ounces were injected; a faint and but momentary indication of rallying was induced. The injection was not repeated, and the woman died in three days afterwards.

Dr. Routh also suggests its use in the diarrhœa of children, where fatal exhaustion is threatened. There are most promising indications for its employment under this head. The numbers of children who die from defective nutrition, arising from imperfect power of assimilation, —their aspect years behind the standard of their age,—who drag on, by aid of iron, iodine, and now of cod liver oil, a miserable existence, to be at last prematurely terminated,—in such cases it is not irrational to anticipate that transfusion, when fairly tested, may prove a remedial agent of greater power and efficiency than any we now possess.—*Medico-Chirurgical Transactions*, vol. xxxv.

* Provincial Medical and Surgical Journal.

